# Indirect estimation of the prevalence of spinal muscular atrophy Type I, II, and III in the United States

**DOI:** 10.1186/s13023-017-0724-z

**Published:** 2017-11-28

**Authors:** Cathy Lally, Cynthia Jones, Wildon Farwell, Sandra P. Reyna, Suzanne F. Cook, W. Dana Flanders

**Affiliations:** 1 Rollins School of Public Health, Emory University, Atlanta, GA USA; 2 Biogen, Cambridge, MA USA; 3 Epidemiology Associates LLC, Chapel Hill, NC USA

**Keywords:** Prevalence, Spinal muscular atrophy, Survival

## Abstract

**Background:**

Spinal muscular atrophy (SMA) is a progressive, devastating disease and a leading inherited cause of infant mortality. The limited population-based literature is confined to small regional studies. Estimates of prevalence are needed to characterize the burden of SMA and to understand trends in prevalence by disease type as new treatments become available. The reported estimates of SMA genotype prevalence at birth consistently range from 8.5–10.3 per 100,000 live births, with a mid-range estimate of 9.4 per 100,000. Among infants born with an SMA genotype, it is reported that ~58% will develop SMA Type I, 29% will develop Type II, and 13% will develop Type III, respectively.

**Results:**

Using evidence from peer-reviewed literature for SMA birth prevalence, age at symptom onset, and SMA type-specific survival, and incorporating United States vital statistics, we constructed life tables to estimate prevalence for SMA Types I, II, and III in the United States. We estimated the number of prevalent cases in the US to be 8526, 9429, and 10,333 based on a birth prevalence of 8.5, 9.4, and 10.3, respectively (the lower, midpoint, and upper ends of the reported range). Assuming the midpoint of 9.4 and US-reported survival, the type-specific population prevalence estimates were 1610 for SMA Type I, 3944 for SMA Type II, and 3875 for SMA Type III. Evidence-based estimates of the number of people living with SMA in the United States in the published literature were previously unavailable.

**Conclusions:**

In the absence of a survey or other means to directly estimate prevalence in the US population, estimates can be calculated indirectly using a life table.

**Electronic supplementary material:**

The online version of this article (10.1186/s13023-017-0724-z) contains supplementary material, which is available to authorized users.

## Background

Spinal muscular atrophy (SMA) is an inherited autosomal recessive neuromuscular disorder characterized by degeneration of motor neurons in the spinal cord and lower brain stem, resulting in severe and progressive muscular atrophy and weakness [1, 2]. SMA is caused by deletions or mutations in the *survival motor neuron 1* (*SMN1*) gene, resulting in little to no function in the SMN protein, which is critical for the maintenance of motor neurons [3, 4]. In the absence of a functional *SMN1* gene, the body relies on its homolog gene called *SMN2* to produce SMN protein. The severity of SMA is associated with the number of *SMN2* gene copies present [5, 6]. Clinically, SMA symptoms range from early infant death in children with SMA Type I to mild weakness in adults with SMA Type IV [7]. Specifically, children with SMA Type I, the most severe life-threatening form, produce very little SMN protein and do not achieve the ability to sit without support or typically live beyond 2 years of age without respiratory support [8]. Individuals with SMA Types II and III produce greater amounts of SMN protein and have less severe but still clinically significant forms of SMA.

SMA is a progressive and devastating disease [1, 6, 8–10] and the leading cause of infant mortality from a single gene disorder [6, 11]. With advances in understanding of the genetic basis of SMA, potential drug strategies include replacement or correction of the mutated *SMN1* gene, modulation of the low-functioning *SMN2* “back-up gene” that is unique to humans, neuroprotection of the motor neurons affected by loss of SMN protein, and muscle protection to prevent or restore loss of muscle function in SMA [6, 12]. The US Food and Drug Administration approved the first treatment for SMA in December 2016 [13]. As new treatments continue to be developed, there is an increased need for robust epidemiologic data to aid in the understanding of disease trends, inform policy regarding allocation of health care resources, anticipate future health care service needs, and support SMA advocacy efforts.

Specifically, as new treatments continue to become available, prevalence estimates are needed to characterize the changing burden of SMA and to understand trends in prevalence by type of SMA. Means of directly estimating the population prevalence include door-to-door surveys, analysis of hospital and clinic records, and systematic surveillance methods. Currently, there are no known mandatory surveillance systems (eg, newborn screening); thus, case reporting to inform the understanding of the size of the SMA population is likely to be incomplete. Studies have provided direct estimates, but only for small populations. Direct estimates of SMA for large populations would require significant resources.

Indirect estimation of SMA prevalence can be informative and cost effective by using available published data [14]. This approach can characterize prevalence of carrier status at birth, but does not directly estimate SMA prevalence in the population as a whole. Despite the limitations of estimates based on SMA carrier status, they can be used to estimate SMA birth prevalence. SMA population prevalence can then be indirectly estimated using SMA birth prevalence and SMA type-specific survival estimates. Available estimates of SMA birth prevalence have limitations. Some suggest that these reported estimates tend to be lower than those expected based on a projection from carrier status [15]. Recent studies of directly observed incidence (eg, incidence of diagnosed disease) that can be used to estimate prevalence indirectly tend to be regionally limited [16, 17] and report different carrier frequencies by geography [15, 18, 19]. Most published studies concerning prevalence and incidence do not provide estimates of survival. Geographical variation in treatment patterns and outcomes is not systematically documented and therefore precludes characterization of expected survival within the population [20–23]. Recognized phenotypes have different characteristics, including symptom onset that ranges from shortly after birth to months or years later [24, 25], as well as very different survival times. This heterogeneity adds to the complexity of prevalence estimation.

The objective of the current study was to estimate the prevalence of people who have been diagnosed and are living with SMA Types I, II, and III in the United States. We used an indirect approach, combining (1) evidence from peer-reviewed literature that provide estimates of the prevalence of an SMA genotype at birth, (2) age at symptom onset, and (3) survival. These estimates were incorporated with US vital statistics and used to construct life tables that provides prevalence estimates for SMA in the United States.

## Methods

Indirect estimation using a life table method was employed to estimate the age- and type-specific prevalence of SMA. The goal was to estimate the total number of people living with symptomatic SMA Types I, II, and III in the United States. Current US age-specific population projections for the year 2016 were obtained from the Centers for Disease Control and Prevention [26]. Projected age-specific survival probabilities for the United States were obtained from the 2010 US life tables published in the Centers for Disease Control and Prevention’s National Vital Statistics Report [27]. Prevalence estimates were calculated separately by SMA type to account for the differing rate of birth prevalence and estimated survival for each type. This method relied on the following 5 types of information. (1) Birth prevalence: here, “birth prevalence” is the proportion of newborns with an SMA genotype. We assumed that each baby born alive with an SMA genotype would eventually be recognized as having an SMA phenotype. We used a reported range of 8.5–10.3 [16, 19, 28, 29] per 100,000 live births, with 9.4 as a midpoint assessment of birth prevalence for SMA Types I, II, and III based on studies that reported population-based estimates of birth prevalence using contemporary case definitions and genetic confirmation, as well as clearly reported numerator and denominator for analysis. We assigned type-specific birth prevalence based on the distribution of SMA types in the published literature [16, 19, 28, 29]. The type-specific distribution estimates used were 58%, 29%, and 13% for SMA Types I, II, and III, respectively [24]. (2) Disease onset: the estimated age of reported symptom onset and diagnosis (as a proxy for disease onset) from the literature for SMA Types I and II was <1 year. For SMA Type III, the earliest age at onset was in the second year, implying cases were not observed at birth or 1 year of age and thus no onset during this interval. [30] (3) SMA survival from birth: for SMA Types I and II, we used available survival estimates from the literature for studies in which survival was reported. We note that a limited body of literature was available and reflected large variation in standards of care and survival. [25] If >1 published estimate was available for a given age, a weighted average of published survival probabilities was used. For these types of SMA, the survival probability for the years not directly observed was interpolated based on periods with available probabilities, by assuming that the 1-year survival probability was constant. For SMA Types I and II, survival estimates were not reported in the literature beyond 20 and 40 years of age, respectively. For ages where survival estimates were unavailable in the literature, it was taken to be nearly 0 (1 × 10^−6^) at and after the ages where available clinical evidence suggested an absence of living cases: 25 years for SMA Type I and 50 years for SMA Type II. For SMA Type III, survival was assumed to be that of the US population. (4) Age-specific survival estimates for the US population by 1-year age groups were taken from the most recent available life table estimates published in the National Vital Statistics Report [27]. (5) Number of persons in the population by age: age-specific (1-year age group) national population projections for the year 2016 were taken from the Centers for Disease Control and Prevention [26]. These population estimates were used to estimate the number of persons with SMA at each year of age.

Additional files 1, 2, 3, and 4 provide details of our calculations, such as the probability of having the diagnosis given survival through a specific age. This probability was multiplied by the US population at each given age to estimate number of people living with each type of SMA for each 1-year age group. The estimated number of prevalent cases of each SMA type was calculated using the sum of the number living with SMA for 0–100+ years of age.

## Results

### Birth prevalence

The estimates of SMA genotype prevalence at birth reported in the literature are consistently in a range from 8.5–10.3 per 100,000 live births [16, 28] or ~1 per 10,000 live births globally (Additional file 1: Table S1). Among infants born with an SMA genotype, it is reported that ~58% will develop SMA Type I, 29% will develop SMA Type II, and 13% will develop SMA Type III [24]. SMA Types 0 and IV are rarely observed [24].

### Survival estimates

For SMA Type I, we reviewed 4 papers from the United States that provide survival estimates for patients with SMA Type I (Additional file 2: Table S2) [9, 31–33]. Survival probabilities across the 4 studies ranged from 37-94% at 1 year and 31–87% at 2 years. Some studies reported survival probabilities at the following other time points: 26–72% [34] at 4 years and 8–50% at 10 years [35–37]. One study utilizing family-reported data found an 18% survival probability at 20 years of age [32]. The populations studied differed substantially in the use of respiratory support. Lemoine et al. (2012) found longer survival among patients for whom caregivers chose to provide noninvasive ventilation at night and daytime sleep and cough assist at least twice a day compared with patients without this support [31]. Oskoui et al. (2007) compared an earlier cohort of patients (1980–1994), an era before respiratory support became the standard of care, to a later cohort (1995–2006); significantly lower survival was observed in the earlier cohort than in the later cohort, a finding confirmed in later studies [32]. It is unknown how many patients with SMA in the United States receive respiratory support. Neither Finkel et al. nor Mannaa et al. reported survival according to use (actual or expected) of respiratory support [9, 33]. Variation across studies also can be attributed to differences in data sources and capture; study periods before, during, or after more frequent use of respiratory support; study duration; and sample size.

Only 1 study reported survival estimate for SMA Type II in the United States. Mannaa et al. (2009) reported US survival for SMA Types I, II, and III (Additional file 3: Table S3) [33]. The survival of patients with SMA Type II was 100% at 1, 2, and 4 years of age. Beyond 4 years of age, the survival was 82% at 10 years and was unchanged at 15 years of age when the study terminated. Because we found only 1 study in the United States detailing survival for SMA Type II, we examined relevant studies in other countries [33, 35, 37] and found similar estimates to Mannaa et al. [33]. Details can be found in Additional files 1, 2, and 3.

For SMA Type III, the literature reports a normal life expectancy (Additional file 3: Table S3) [33–37].

To evaluate the sensitivity of the estimated prevalence to the different survival rates reported in the literature and differing survival rates reported for patients treated versus untreated, we used 2 main values for survival: pooled estimates based on US populations only and pooled estimates based on US, European, and Australian populations. Reported survival estimates are lower in Europe, where respiratory support was less often noted, and result in lower prevalence estimates.

### Prevalent cases in the US

Because we found a range of birth prevalence estimates in the literature, we estimated the 2016 US population prevalence of SMA Types I, II, and III using 3 birth prevalence estimates: either lower birth prevalence (8.5), higher birth prevalence (10.3), or the midpoint of the reported birth prevalence (9.4, an average of the 8.5–10.3 range of prevalence). We estimated the number of prevalent cases in the US to be 8526, 9429, and 10,333, based on a reported birth prevalence of 8.5, 9.4, and 10.3, respectively, and US-reported survival estimates (Tables 1, 2, and 3). Assuming the midpoint of 9.4 and US-reported survival, the type-specific numbers of prevalent cases were 1610 for SMA Type I, 3944 for SMA Type II, and 3875 for SMA Type III (Table 2). Type-specific estimates assuming the lowest- and highest-reported birth prevalence and estimates assuming a lower reported survival are reported in Tables 1, 2, and 3. The overall range of the number of people living with SMA Types I, II, and III in the United States based on reported birth prevalence and variability in reported survival estimates was 7901–10,333.

**Table 1:** Estimated 2016 US SMA prevalence by type assuming a birth prevalence of 8.5 per 100,000

SMA type	Estimated prevalence^a^	Estimated prevalence^b^
I	1455	1067
II	3567	3330
III	3504	3504
All	8526	7901

*SMA* spinal muscular atrophy

^a^Used published survival estimates from US literature only

^b^Used combined published survival estimates from the United States, Europe, and Australia

**Table 2:** Estimated 2016 US prevalence of SMA by type assuming a birth prevalence of 9.4 per 100,000

SMA type	Estimated prevalence^a^	Estimated prevalence^b^
I	1610	1180
II	3944	3682
III	3875	3875
All	9429	8737

*SMA* spinal muscular atrophy

^a^Used published survival estimates from US literature only

^b^Used combined published survival estimates from the United States, Europe, and Australia

**Table 3:** Estimated 2016 US prevalence of SMA by type assuming a birth prevalence of 10.3 per 100,000

SMA type	Estimated prevalence^a^	Estimated prevalence^b^
I	1764	1293
II	4322	4035
III	4247	4247
All	10,333	9575

*SMA* spinal muscular atrophy

^a^Used published survival estimates from US literature only

^b^Used combined published survival estimates from the United States, Europe, and Australia

## Discussion

Utilizing survival estimates from published US studies, we estimated that 8526–10,333 individuals with SMA Types I, II, and III are living in the United States in 2016. Approximately 1455–1764 of these people have SMA Type I, 3567–4322 have SMA Type II, and 3504–4247 have SMA Type III. Three estimates were calculated based on the highest- and lowest-reported birth prevalence and 1 was based on an average of the 2 estimates. It should be noted that the average of 9.4 per 100,000 live births is closest to the estimates generated from US birth prevalence (Prior 2010 and Sugarman 2012), yielding an estimate of 9429 cases [19, 29]. Estimates calculated using survival probabilities reported from the United States generally suggest a higher prevalence than those estimates calculated using survival probabilities reported from Europe and Australia. Using combined survival probabilities from the United States, Europe, and Australia, we estimated that 7501–9575 individuals with SMA are living in the United States in 2016.

The distribution of prevalent cases we report, with more people having SMA Types II and III and fewer having SMA Type I, is expected because of the high case fatality among patients with SMA Type I. With advances in treatment and greater use of the recommended standard of care, prevalence, especially among patients with SMA Type I, is expected to increase over time.

The prevalence estimates are based on the US population estimates and US lifetables for survival. Therefore, the estimates cannot be extrapolated to other countries. However, the methods used to calculate the estimates can be applied to country-specific population estimates and lifetable estimates to derive estimates of the prevalence of SMA in other countries. It may be necessary to use different SMA survival estimates in countries where the treatment for SMA differs from that in the US.

### Limitations

To approximate the number of patients at older ages, survival was assumed to be negligible after 25 years in patients with SMA Type I and 50 years in patients with SMA Type II. Second, survival expected for each year of life among those with SMA have not been reported. To estimate survival, we assumed a constant decline in survival between reporting periods (eg, constant decline in survival between 2 and 4, 4 and 10, 10 and 20 years of age). Third, the standard of care demonstrates great variability so that survival in different periods is probably not constant. To the extent survival will likely increase in the future, the numbers of people living with SMA will tend to increase. Selection bias may be of concern in the published studies. If patients and their families who receive more aggressive care were more likely to participate, overestimation of survival could have resulted. Similar considerations hold for clinicians if those providing more aggressive care were more likely to conduct or participate in such studies.

## Conclusions

Estimates of the number of people living with SMA in the United States in the published literature were previously unavailable. In the absence of a survey or other means to directly estimate prevalence in the US population, we used an indirect method. By utilizing available published estimates of genotype prevalence at birth, age of disease onset, and subsequent survival, we were able to estimate the SMA population for each year of age and subsequently for the entire population.

## Additional files

Additional file 1: Table S1. Summary of contemporary published estimates of SMA birth prevalence. Table showing summary of contemporary published estimates of SMA birth prevalence. (DOCX 23 kb)

Additional file 2: Table S2. Summary of survival probabilities for patients with SMA Type I in the United States. Table showing summary of survival probabilities for patients with SMA Type I in the United States. (DOCX 20 kb)

Additional file 3: Table S3. Summary of survival probabilities for patients with SMA Type II in the United States. Table showing summary of survival probabilities for patients with SMA Type II in the United States. (DOCX 14 kb)

Additional file 4: Table S4. Methods. Table showing methods. (DOCX 16 kb)

## Abbreviations

SMA: spinal muscular atrophy
SMN: survival motor neuron
