# Supplementary material for: Indirect estimation of the prevalence of spinal muscular atrophy Type I, II, and III in the United States
Source: Orphanet J Rare Dis. 2017 Nov 28;12:175. doi: 10.1186/s13023-017-0724-z (PMC5704427; doi:10.1186/s13023-017-0724-z)
Supplement: Supplementary file 1 — Summary of contemporary published estimates of SMA birth prevalence. Table showing summary of contemporary published estimates of SMA birth prevalence. (DOCX 23 kb) [file 13023_2017_724_MOESM1_ESM.docx]

**Additional file 1: Table S1** Summary of contemporary published estimates of SMA birth prevalence^a^

| Author Country |  | | SMA type | | | |
| --- | --- | --- | --- | --- | --- | --- |
|  |  |  | All | I | II | III |
|  | Time period | No. of live births | Prevalence per 100,000 live births (95% CI) | | | |
| United States |  |  |  |  |  |  |
| Prior et al [[1](#_ENREF_1)]  United States | NR | NR | 10.0 (2.6–22.1) | NR | NR | NR |
| Sugarman et al [[2](#_ENREF_2)]^b^  United States | May 2008–May 2009 | NR | 9.1 (0.45–44.8) | NR | NR | NR |
| Other geographies |  |  |  |  |  |  |
| Arkblad et al [[3](#_ENREF_3)] Sweden | 1980–2006 | 531,746 | 8.5 (6.2–11.3) | 3.6 (2.3–5.6) | 2.1 (1.2–3.7) | 2.8 (1.7–4.7) |
| Jedrzejowska et al [[4](#_ENREF_4)] Poland | 1998–2005 | 2,963,783 | 10.3 (9.2–11.5) | 7.1 (6.2–8.1) | 1.3 (0.9–1.7) | 2.0 (1.5–2.5) |

*NR* not reported; *SMA* spinal muscular atrophy

^a^ The studies were selected that reported population-based estimates of birth prevalence using contemporary case definitions and genetic confirmation, as well as clearly reported numerator and denominator for analysis.

^b^Calculated prevalence based on carrier status in a large population

**References**

1. Prior TW, Snyder PJ, Rink BD, Pearl DK, Pyatt RE, Mihal DC, et al. Newborn and carrier screening for spinal muscular atrophy. Am J Med Genet A. 2010;152A:1608–16.

2. Sugarman EA, Nagan N, Zhu H, Akmaev VR, Zhou Z, Rohlfs EM, et al. Pan-ethnic carrier screening and prenatal diagnosis for spinal muscular atrophy: clinical laboratory analysis of >72,400 specimens. Eur J Hum Genet. 2012;20:27–32.

3. Arkblad E, Tulinius M, Kroksmark AK, Henricsson M, Darin N. A population-based study of genotypic and phenotypic variability in children with spinal muscular atrophy. Acta Paediatr. 2009;98:865–72.

4. Jedrzejowska M, Milewski M, Zimowski J, Zagozdzon P, Kostera-Pruszczyk A, Borkowska J, et al. Incidence of spinal muscular atrophy in Poland–more frequent than predicted? Neuroepidemiology. 2010;34:152–7.
