# Supplementary material for: Indirect estimation of the prevalence of spinal muscular atrophy Type I, II, and III in the United States
Source: Orphanet J Rare Dis. 2017 Nov 28;12:175. doi: 10.1186/s13023-017-0724-z (PMC5704427; doi:10.1186/s13023-017-0724-z)
Supplement: Supplementary file 2 — Summary of survival probabilities for patients with SMA Type I in the United States. Table showing summary of survival probabilities for patients with SMA Type I in the United States. (DOCX 20 kb) [file 13023_2017_724_MOESM2_ESM.docx]

**Additional file 2: Table S2** Summary of survival probabilities for patients with SMA Type I in the United States

| Author Time period, location | N | Survival probability, %^a^ | | | | |  |
| --- | --- | --- | --- | --- | --- | --- | --- |
|  |  | Age, y | | | | |  |
|  |  | 1 | 2 | 4 | 10 | 20 | |
| Finkel et al [[1](#_ENREF_1)]  2005–2009, United States (New York, NY; Boston, MA; Philadelphia, PA) | Type IB, 18 | 75 | NR | NR | NR |  | |
|  | Type IC, 16 | ~94 | ~87 | NR | NR |  |  |
| Lemoine et al [[2](#_ENREF_2)] 2002–2009, United States (UT) | 49 |  |  |  | NR | NR | |
|  | No NIV, 26 | ~58 | ~44 | ~35 |  |  |  |
|  | NIV, 23 | ~72 | ~72 | ~72 |  |  |  |
| Oskoui et al [[3](#_ENREF_3)] 1980–1994 birth cohort, international, mostly North America | 65 | 37 | 31 | 26 | 25 | 18.1 | |
| Oskoui et al [[3](#_ENREF_3)] 1995–2006 birth cohort, international, mostly North America | 78 | 79 | 74 | 65 | 50 |  | |
| Mannaa et al [[4](#_ENREF_4)]  1989–2005, United States (Cincinnati, OH)^b^ | 15 | 92 | 72 | 62 | 8 | NR | |

*NR* not reported; *NIV* noninvasive ventilation; *SMA* spinal muscular atrophy

^a^Survival probabilities for all studies calculated using the Kaplan–Meier method

^b^Survival probabilities from Fig. 1 in Mannaa et al [[4](#_ENREF_4)]

**References**

1. Finkel RS, McDermott MP, Kaufmann P, Darras BT, Chung WK, Sproule DM, et al. Observational study of spinal muscular atrophy type I and implications for clinical trials. Neurology. 2014;83:810–7.

2. Lemoine TJ, Swoboda KJ, Bratton SL, Holubkov R, Mundorff M, Srivastava R. Spinal muscular atrophy type 1: are proactive respiratory interventions associated with longer survival? Pediatr Crit Care Med. 2012;13:e161–5.

3. Oskoui M, Levy G, Garland CJ, Gray JM, O'Hagen J, De Vivo DC, et al. The changing natural history of spinal muscular atrophy type 1. Neurology. 2007;69:1931–6.

4. Mannaa MM, Kalra M, Wong B, Cohen AP, Amin RS. Survival probabilities of patients with childhood spinal muscle atrophy. J Clin Neuromuscul Dis. 2009;10:85–9.
