# Supplementary material for: Indirect estimation of the prevalence of spinal muscular atrophy Type I, II, and III in the United States
Source: Orphanet J Rare Dis. 2017 Nov 28;12:175. doi: 10.1186/s13023-017-0724-z (PMC5704427; doi:10.1186/s13023-017-0724-z)
Supplement: Supplementary file 3 — Summary of survival probabilities for patients with SMA Type II in the United States. Table showing summary of survival probabilities for patients with SMA Type II in the United States. (DOCX 14 kb) [file 13023_2017_724_MOESM3_ESM.docx]

**Additional file 3: Table S3** Summary of survival probabilities for patients with SMA Type II in the United States

|  | | Survival probability, %^a^ | | | | |
| --- | --- | --- | --- | --- | --- | --- |
| Author Time period, location | N | Age, y | | | | |
|  |  | 1 | 2 | 4 | 10 | 20 |
| Type II |  |  |  |  |  |  |
| Mannaa et al [[1](#_ENREF_1)]  1989–2005, United States (Cincinnati, OH)^b^ | 15 | 92 | 72 | 62 | 8 | NR |

*NR* not reported; *SMA* spinal muscular atrophy

^a^Survival probabilities for all studies calculated using the Kaplan–Meier method

^b^Survival probabilities from Fig. 1 in Mannaa et al [[1](#_ENREF_1)]

**Reference**

1. Mannaa MM, Kalra M, Wong B, Cohen AP, Amin RS. Survival probabilities of patients with childhood spinal muscle atrophy. J Clin Neuromuscul Dis. 2009;10:85–9.
