# Supplementary material for: Indirect estimation of the prevalence of spinal muscular atrophy Type I, II, and III in the United States
Source: Orphanet J Rare Dis. 2017 Nov 28;12:175. doi: 10.1186/s13023-017-0724-z (PMC5704427; doi:10.1186/s13023-017-0724-z)
Supplement: Supplementary file 4 — Methods. Table showing methods. (DOCX 16 kb) [file 13023_2017_724_MOESM4_ESM.docx]

**Additional file 4: Table S4** Methods

| Notation and description of information | Notation |
| --- | --- |
| Has SMA genotype in form eventually expressed if survive to usual age at diagnosis (indicator variable) | G=1, 0 |
| Proportion of newborns with genetic abnormality who eventually express phenotype | P(G=1) |
| Survive to age A (indicator variable) | S_A_=1, 0 |
| Survival to age A from birth of those with genetic abnormality, | P(S_A_=1\|G=1) |
| Usual age at diagnosis | D_SMA_ |
| Proportion of US population surviving to age A (life table; P(S_A_=1\|G=0) approximated by P(S_A_=1) ) | P(S_A_=1\|G=0) |
| No. of people at age A in the United States in 2014 (1-year age groups) | N_A_ |
| No. of people with diagnosed SMA in the United States in 2014 at age A (1-year age groups) | N_A,S_ |

*SMA* spinal muscular atrophy

To estimate the age-specific prevalence for each type of SMA, we use a life table approach, first calculating conditional probabilities of having the diagnosis given survival through age A (S_A_=1). We assume that people are not diagnosed before the usual age at diagnosis (D_SMA_) and that diagnosis occurs at age D_SMA_ for those with G=1:

P(diagnosis|S_A_=1) = 0 for A < D_SMA_ ; and P(diagnosis|S_A_=1, G=1) = 1 for A ≥ D_SMA_

We assume that survival is normal before diagnosis (in particular, for A ≤ D_SMA_):

P(S_A_=1|G=1) = (P S_A_=1|G=0) = P(S_A_=1) for A ≤ D_SMA_.

Using rules of conditional probabilities we have the following probabilities for A > D_SMA_

P(diagnosis|S_A_=1) = P(S_A_=1|G=1) P(G=1) / P(S_A_=1)

where P(diagnosis) = P(G=1), because we assume that all with G=1 are diagnosed at age D_SMA_.

Notes:

1. These conditional probabilities can be interpreted as the probability that someone has the diagnosis if selected at random from the subgroup alive at age A
2. Much of the needed information is not directly available from the literature, so we interpolated and supplemented based on clinical judgement as described below and in main text
3. Age-specific survival for those with the disease depends on type. For SMA Type I, diminished survival was assumed to begin at birth for all with the genetic abnormality. For SMA Type II, meaningfully diminished survival was assumed to begin at 3 years of age for all with the genetic abnormality. For SMA Type III, those with disease were assumed to have normal survival before and after diagnosis
4. In the absence of published estimates of age- and type-specific survival at a particular age A, but when published estimates are available for age A_1_ < A and at A_2_ > A, we interpolated. For example, if the literature indicates that, after having been diagnosed with SMA, P(S_A1_=1|G=1) = s1 and P(S_A2_=1|G=1) = s2 for 2 ages A1 < A2, then assumed a steadily decreasing survival between age A1 and A2, and interpolated the survival between these ages. The proportion s’ surviving from age A to A + 1 is interpolated as:

s’ = [P(S_A2_=1|G=1) / P(S_A1_=1|G=1)]^(1/(A2-A1)),

and the overall survival at age *j*, for A1 < *j* < A2 as:

s_j_ = P(S_A1_=1|G=1) ×{[P(S_A2_=1|G=1)/ P(S_A1_=1|G=1)]^(1/(A2-A1))}^(j-A1)

With this approach, the interpolated estimates agree with published estimates for the 2 ages A1 and A2 when published estimates are available, and decrease smoothly between these 2 ages

In the absence of age- and type-specific estimates of survival at older ages, we supplement with clinical and epidemiological judgment. Specifically, for SMA Type I we found no published reports about survival after ~20 years of age. However, clinical judgment suggested that no persons with SMA Type I survive beyond 25 years of age, thus we set P(S_A_=1|G=1) to a small number close to 0 (1.0×10^–9^) at 25 years of age. For ages between 20 and 25 years, we interpolated as in (3) above. Above 25 years of age, we took the 1-year survival to be s’=0.0233, as calculated in (3). For SMA Type II we found no published reports about survival after ~40 years of age. However, clinical judgment suggested that no persons with SMA Type II survive beyond 50 years of age, thus we set P(S_A_=1|G=1) to a small number close to 0 (1.0×10^–9^) at 50 years of age. For ages between 40 and 50 years, we interpolated as in (3) above. Above 50 years of age, we took the 1-year survival to be s’=0.1895, as calculated in (3)

Using these equations, we obtained the estimator:

N_A,S_ = N_A_×P(diagnosis|S_A_=1)

Finally, the number of cases (SMA) is estimated by:

$SMA= \sum_{A=0}^{A=100} N_{A,S}$
